# A new mark-recapture approach for abundance estimation of social species

**DOI:** 10.1371/journal.pone.0208726

**Published:** 2018-12-20

**Authors:** Jena R. Hickey, Rahel Sollmann

**Affiliations:** 1 International Gorilla Conservation Programme, Musanze, Rwanda; 2 Department of Wildlife, Fish, and Conservation Biology, University of California Davis, Davis, CA, United States of America; University of Hong Kong, HONG KONG

## Abstract

Accurate estimates of population abundance are a critical component of species conservation efforts in order to monitor the potential recovery of populations. Capture-mark-recapture (CMR) is a widely used approach to estimate population abundance, yet social species moving in groups violate the assumption of CMR approaches that all individuals in the population are detected independently. We developed a closed CMR model that addresses an important characteristic of group-living species–that individual-detection probability typically is conditional on group detection. Henceforth termed the Two-Step model, this approach first estimates group-detection probability and then–conditional on group detection–estimates individual-detection probability for individuals within detected groups. Overall abundance is estimated assuming that undetected groups have the same average group size as detected groups. We compared the performance of this Two-Step CMR model to a conventional (One-Step) closed CMR model that ignored group structure. We assessed model sensitivity to variation in both group- and individual-detection probability. Both models returned overall unbiased estimates of abundance, but the One-Step model returned deceptively narrow Bayesian confidence intervals (BCI) that failed to encompass the correct population abundance an average 52% of the time. Contrary, under the Two-Step model, CI coverage was on average 96%. Both models had similar root mean squared errors (RMSE), except for scenarios with low group detection probability, where the Two-Step model had much lower RMSE. For illustration with a real data set, we applied the Two-Step and regular model to non-invasive genetic capture-recapture data of mountain gorillas (*Gorilla beringei beringei*). As with simulations, abundance estimates under both models were similar, but the Two-Step model estimate had a wider confidence interval. Results support using the Two-Step model for species living in constant groups, particularly when group detection probability is low, to reduce risk of bias and adequately portray uncertainty in abundance estimates. Important sources of variation in detection need to be incorporated into the Two-Step model when applying it to field data.

## Introduction

Robust estimates of species population abundance are necessary for understanding baseline conditions, gauging population trends, assessing the success of conservation actions, and developing management strategies. Closed population Capture-mark-recapture (CMR) is considered the gold standard to estimating wildlife abundance while accounting for our imperfect ability to detect all individuals in a population [1, 2, 3, 4, 5, 6]. Pollock [1] and Otis *et al*. [2] described early versions of CMR and, due to its pervasive use by population ecologists, Pollock [7] reviewed these approaches a short 5 years later. Since then, numerous review papers have described various closed CMR approaches to population estimation [8, 9] with several important additions (e.g. [10, 11, 12]) and cautions regarding the consequences of violating assumptions of CMR (e.g. [3]). CMR requires individual-level detection histories, which were originally limited to live-trapping studies, but with advances in non-invasive survey methodology, can now be obtained from non-invasive genetic markers found in hair or feces of target species [13, 14, 15].

Social species provide a particular challenge when applying CMR approaches because the nature of group living typically violates a primary assumption of CMR–that individuals are detected independently of each other–which introduces a special kind of capture heterogeneity. The distributions of individuals of group-living species are clumpy; hence, in most cases, detection of the individual is dependent on first detecting the group [16]. In such cases, individual-level detection probability *p*_*i*_ = 0 for individuals belonging to undetected groups and *p*_*i*_>0 for individuals belonging to detected groups, thereby representing a special case of heterogeneity in detection probability among individuals in the population. Several approaches have been developed to deal with capture heterogeneity. These include the jackknife heterogeneity model [17, 2] as well as finite mixture [18] or two-innate-rates (TIRM) models [13] for unobservable heterogeneity; logistic-regression type models including individual covariates [19, 20, 21]; and spatial capture-recapture models, which account for detection heterogeneity due to different exposure to sampling [22]. Thus far, there is no CMR approach that explicitly describes the dependence of *p*_*i*_ on group detection that is characteristic of many group-living species. Failure to account for heterogeneity in detection leads to overestimating parameter precision and potentially biased parameter estimates [23]. Over time, biased abundance estimates coupled with falsely narrow confidence intervals (CIs) could lead to erroneous conclusions about population trends, whether they be false estimates of growth or decline [24], and consequently, misguided conservation actions.

Mountain gorillas (*Gorilla beringei beringei*) serve as an excellent model species to develop a capture-recapture approach accounting for lack of independence among individual detections due to group living. Mountain gorillas live in fairly stable family groups and they are of such high conservation concern that for the last several decades, survey efforts expending enormous financial and staff resources have been conducted approximately every 5–10 years to estimate their population abundance [24, 25]. Although unhabituated gorillas are difficult to detect due to thick vegetation, rough terrain, and elusive behavior, non-invasive genetic markers extracted from fecal samples found in gorilla nests provide a way to survey mountain gorillas, even if they are unhabituated to humans [24]. In established protocols for surveying mountain gorilla populations [25, 26, 27], individual gorilla nests are sampled only upon detection of the entire group’s nest site, thereby underscoring the dependence of individual detections on group detection.

Here, we present a Two-Step closed CMR approach (henceforth, Two-Step model) that accounts for the dependent nature of detection for group-living species by estimating both a group-detection probability and then, conditional on group detection, an individual-detection probability. Using simulations informed by a field dataset on mountain gorillas, we compare the performance of the conventional closed CMR model (henceforth, One-Step model) that assumed all individuals have the same detection probability with our prototype Two-Step CMR model. We demonstrate application of the Two-Step model by re-analyzing data from the 2011 mountain gorilla survey [26]. Finally, we describe the strengths and weaknesses of the models and offer recommendations for future model development. Though motivated by mountain gorillas, the Two-Step CMR model is applicable to a wide range of group-living taxa such as lions, wolves, elephants, or wild pigs.

## Methods

### Simulations

We simulated populations of group-living animals and generated capture histories for these animals using program R version 3.4.0 [28]. Because model development was motivated by estimating population size for the group-living mountain gorilla, data from the 2010 Virunga population survey of mountain gorillas [25, 27] generally informed the setup of our population simulations. For example, Gray *et al*. [25] reported 36 groups with mean group size of 12.5, and in our simulations each population consisted of 40 groups with group size generated as a zero-truncated Poisson random variable with a mean of 13 (for full description of generation of simulated populations see R-code in S1 Script).

Capture histories were simulated reflecting a field-sampling design for mountain gorillas [26]. Individuals in gorilla groups construct new nests each night and they usually defecate in the nests prior to departing in the morning [29]. Areas encompassing all nests from a gorilla group constructed on a given night are termed nest sites. Researchers searched the entire study area for these nest sites in what they termed ‘sweeps’. Upon observing a gorilla nest site during a given sweep and collecting fecal samples from each nest, teams followed connecting fresh gorilla trails through the forest aiming to find 3 nest sites per gorilla group [26]. Hence, our simulated capture–recapture data were based on 2 trials (‘sweeps’) to detect groups, yielding two-trial group-level detection histories; and 3 sub-trials (nest sites) to detect individuals within those observed groups, yielding three-trial individual-level detection histories for each ‘sweep’. If a group was not detected during a given sweep, all individuals in that group had all-zero detection histories.

We analyzed the generated data with two hierarchical CMR models. For the conventional CMR model (i.e., ignoring group structure of the population), we followed current practice of analyzing the gorilla survey data [26] and constructed individual-level encounter histories with sweeps constituting sampling occasions *K*. Within a sweep, this condenses individual detections across multiple nest sites to a single binary observation, *y*_*ik*_, modeled as a Bernoulli random variable with success probability (i.e., individual detection probability) *p*. To estimate abundance *N*, including those individuals never detected, we employed data augmentation [30] where the observed detection histories are augmented with a large number *M* of all-zero detection histories, and the model estimated how many of these augmented individuals were actually in the population but never detected (for full One-Step CMR Model description see JAGS code in S2 Script).

In contrast, the novel Two-Step model first used binary group-level detection with sweeps as occasions *K* to estimate the number of groups (including groups never observed) *G*. To estimate *G*, we used data augmentation, analogous to estimating *N* in the conventional CMR model. To estimate group size *m*_*g*_, we made use of the individual-level detection histories with *T* nest sites as occasions, building on the standard multinomial formulation of closed capture-recapture models [31]. Following Clement et al. [32], we describe group size as a zero-truncated Poisson random variable. The collection of individual detection histories from an observed group *i*, ***ω***_***i***_, can be described as conditional multinomial random variable. As a simple example, for *K* = 2 sweeps and *T* = 1 nest site, ***ω***_***i***_ is a vector containing the number of individuals detected in sweep 1 but not in sweep 2 (detection history “10”), the number detected in sweep 2 but not sweep 1 (“01”), and the number detected in both sweeps (“11”). The number of individuals never detected (“00”) is unobserved. Cell probabilities for the multinomial distribution of ***ω***_***i***_ can be derived as p*(1−*p*), (1−*p*)*p, and p*p, where *p* is the individual detection probability in a given sweep. To account for the unobserved detection history “00”, these cell probabilities are divided by the probability of being detected at least once (i.e., conditioned on detection), p* = 1−((1−*p*)*(1−*p*)). This can readily be expanded to any number of *K* occasions. In our simulation, *p* = *p*.*i*, individual-detection probability. We achieve conditioning of individual detection on group detection by multiplying *p*.*i* with the occasion-specific group observations, so that *p*.*i* = 0 during a sweep in which the group was not detected. Finally, to estimate group size, we model total number of observed individuals in group *i*, *n*_*i*_, as a zero-truncated Binomial random variable with sample size *m*_*g*_ and detection probability p*. Group sizes for unobserved groups are generated as random draws from the zero-truncated Poisson distribution of group sizes. We obtained total population size *N* by summing *m*_*g*_ for all groups estimated (by data augmentation) to be part of the population (for full Two-Step CMR Model description see JAGS code in S3 Script). The Two-Step Model returned estimates of the average number of groups, average group size, and total population size; whereas the One-Step Model returned only estimates of total population size.

Note that for the purpose of model development and comparison, we use a simple detection model that does not include individual heterogeneity in detection probability (in data generation or analysis). Both the One-Step and Two-Step models described above can easily be extended to incorporate sources of heterogeneity in *p* (see Discussion).

We performed these simulations for a range of possible values of group level detection, *p*_*g*_, and individual level detection, *p*_*i*_, to test model performance under different detection probabilities. Specifically, we generated group- and individual-level detection histories by holding *p*_*g*_ constant at 0.7 while varying *p*_*i*_ from 0.3 through 0.9 at intervals of 0.2, and vice versa (a constant *p*_*i*_ of 0.7 and *p*_*g*_ of 0.3, 0.5, 0.7, and 0.9). Data from Roy *et al*. [26] suggested that mean detection probabilities of mountain gorillas are approximately 0.7, making this value a reasonable starting point for simulation scenarios. We generated 1000 population data sets and ran both the One-Step and Two-Step CMR models for each of the 8 *p*_*i*_*/p*_*g*_ scenarios. We implemented models in a Bayesian framework using the computer program JAGS [33] through the software R [28] using the jagsUI package [34]. We used uninformative uniform(0,1) priors on all detection probabilities and the data augmentation parameter, and an uninformative uniform(0,30) prior for average group size. Because simulated average group size was 13, this upper bound did not constrain estimates of this parameter (for the One-Step and Two-Step JAGS models see S2 and S3 Scripts, respectively). We compared model performance based on the posterior mean of total abundance *N*, by calculating, across the 1000 simulations, (1) root-mean squared error (RMSE), (2) average bias of the posterior mean, (3) 95% Bayesian Credible Interval (BCI) coverage, and (4) average coefficient of variation (CV).

### Comparison of estimators with real data

To evaluate model performance with real data, which tend to be less ideal than simulated data, we also applied the Two-Step CMR model and the conventional CMR model to noninvasive genetic survey data from unhabituated mountain gorillas collected as described under *Simulations* and in Roy *et al*. [26]. The data set consisted of detections of 179 individuals in 26 groups ranging in observed size from 2 to 17 (mean of 6.9), and 16 solitaries. Solitary mountain gorillas normally occur in mountain gorilla populations. Usually solitaries are adult males that have yet to amass a group of females [35]. In any case, solitaries are substantially more difficult to detect than gorilla groups (e.g. only 1 of 16 solitaries was detected in both ‘sweeps’ [26]).

To demonstrate the added flexibility of the Two-Step model in considering group structure, we applied a slightly modified version, where single individuals have a separate detection probability, *p*_*s*_, their group size is assumed known (i.e., 1), and the number of single individuals in the population is estimated with a separate data augmentation step. Total abundance *N* is the sum of individuals in groups and solitaries. As in the simulations, we used uninformative uniform priors on all parameters; we modeled group size as a Poisson random variable truncated at 2, as minimum group size was 2.

We compared Two-Step model estimates of total abundance with estimates from a conventional CMR model. Roy *et al*. [26] explored multiple CMR models with different sources of variation in detection probability and presented model-averaged estimates of abundance. For our purposes of comparing abundance estimates between the Two-Step and the conventional CMR model, we only applied their [26] most supported model–the null model with no variation in detection probability. We did not engage in model selection or averaging, or compare the Two-Step model to conventional CMR estimates from models accounting for variation in detection. As a result, our estimates under the conventional CMR model differ slightly from those previously published and are not intended as a comparison to Roy et al. [26].

We implemented both models in JAGS, running 3 parallel chains for 50,000 iterations and discarding the first 1,000 as burn-in. According to the Gelman-Rubin statistic, chains for all parameters in the model converged (r-hat<1.1). We present parameter estimates as posterior mean, standard deviation, and 95% BCI.

## Results

### Simulations

Across all scenarios, true abundance ranged from 436 to 606 individuals. Depending on the scenario (i.e. values of *p*_*g*_ and *p*_*i*_), the average proportion of groups detected at least once ranged from 51.3 to 99.9%, and the average proportion of group members detected at least once ranged from 77.8 to 100%. The average posterior mean estimate of abundance ($\hat{N}$) per scenario ranged from 518.9 to 546.6 individuals in the Two-Step and from 521.6 to 556.1 for the One-Step Models.

**Fig 1:**
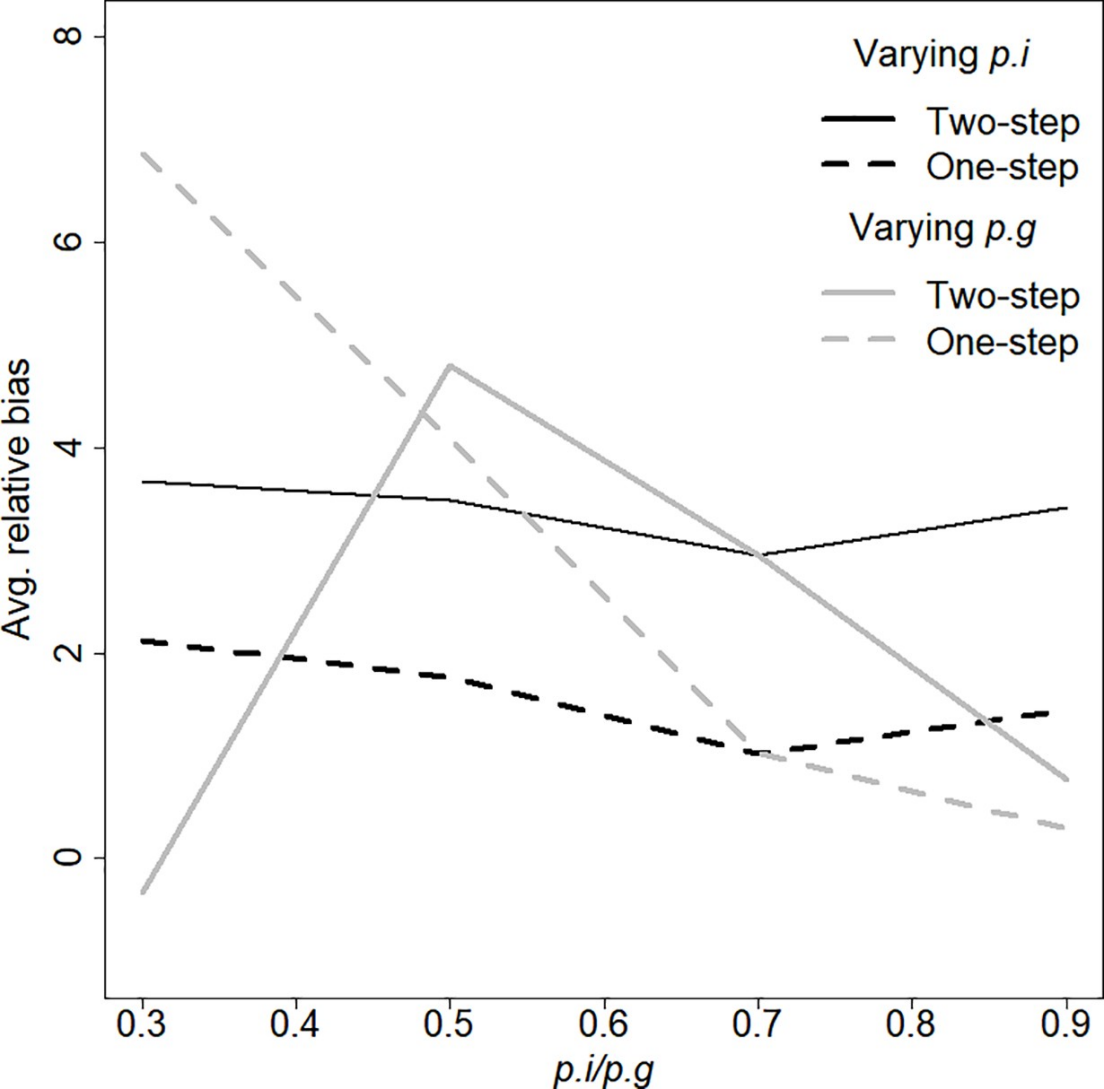
Average relative bias of the posterior mean of abundance ($\hat{N}$) from the Two-Step and the One-Step CMR model over a range of group-detection probabilities (*p*_*g*_) and individual-detection probability (*p*_*i*_). When *p*_*g*_ was varied, *p*_*i*_ was held constant at 0.7, and vice versa.

Average bias in the posterior mean abundance estimates was consistently low to moderate across all combinations of *p*_*i*_ and *p*_*g*_ (ranging from 0% to 5% for the Two-Step and from 0% to 7% for the One-Step model; Fig 1). For constant *p*_*i*_, the One-Step model was sensitive to very low *p*_*g*_ (7% bias at *p*_*g*_ = 0.3) and produced $\hat{N}$ with decreasing bias as *p*_*g*_ increased (Fig 1), whereas the Two-Step model produced most biased estimates of abundance (5% bias) at intermediate levels of *p*_*g*_. With constant *p*_*g*_, bias in both models remained constant across values of *p*_*i*_. For these scenarios, Two-Step model estimates of abundance were consistently more biased that One-Step model estimates, but only by about 2% (Fig 1).

For the Two-Step model, we were able to parse out whether estimation of the number of groups or estimation of group size contributed more to bias in overall abundance estimates. Whereas the average group size was estimated, on average across all scenarios, with <1% bias, estimates of the number of groups had an average bias of 3%, and thus were largely responsible for bias in $\hat{N}$ in the Two-Step model.

**Fig 2:**
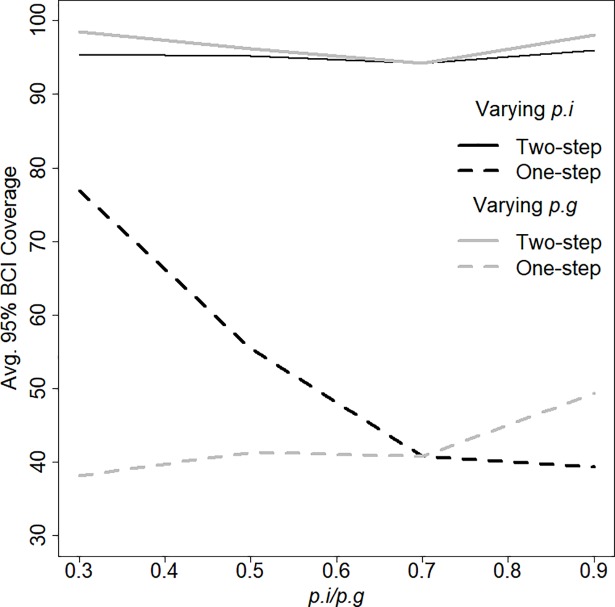
95% Bayesian Credible Interval (BCI) coverage for abundance estimates across 1000 simulated populations for the Two-Step and the One-Step CMR model over a range of group-detection probabilities (*p*_*g*_) and individual-detection probability (*p*_*i*_). When *p*_*g*_ was varied, *p*_*i*_ was held constant at 0.7, and vice versa.

Overall, the Two-Step CMR model performed considerably better in terms of 95% BCI coverage than the conventional model. Two-Step model BCIs encompassed the true population abundance an average of 96% of the time across all scenarios and coverage showed no relationship with varying group or individual detection probability (Fig 2). The One-Step model portrayed false certainty through overly narrow BCIs that encompassed the true abundance, on average, 48% of the time. When *p*_*g*_ was low, coverage of the One-Step model was highest, at 77%, but quickly declined with increasing *p*_*g*_. One-Step coverage was consistently low (between 40 and 50%) across a range of *p*_*i*_ when *p*_*g*_ = 0.7 (Fig 2).

**Fig 3:**
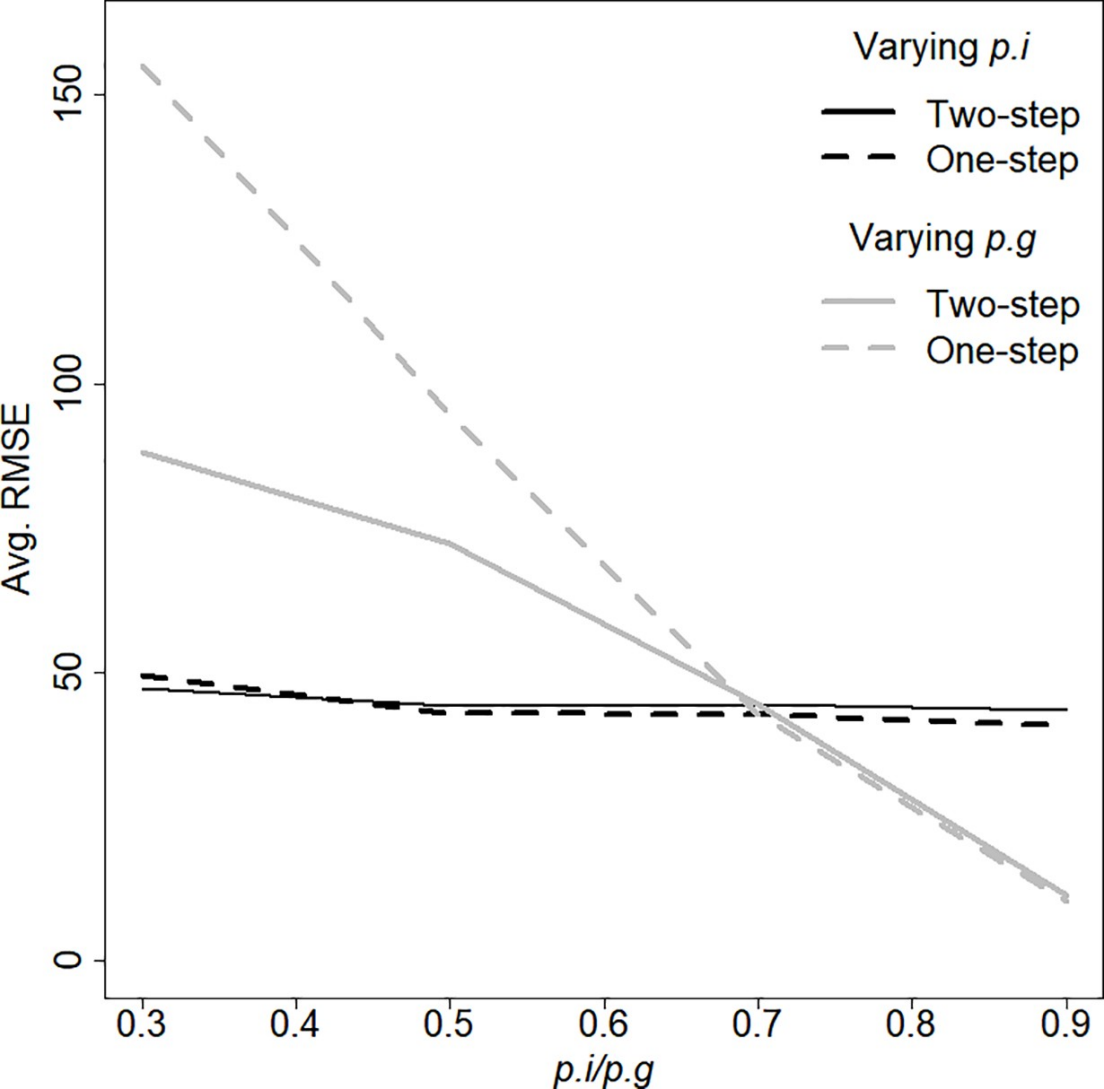
Root mean square error (RMSE) for abundance estimates across 1000 simulated populations for the Two-Step and the One-Step CMR model over a range of group-detection probabilities (*p*_*g*_) and individual-detection probability (*p*_*i*_). When *p*_*g*_ was varied, *p*_*i*_ was held constant at 0.7, and vice versa.

Abundance estimates from the Two-Step model had either smaller or almost identical RMSE compared to the One-Step model. For both models, the RMSE was highest at low group detection probabilities (*p*_*g*_ = 0.3 or 0.5) and decreased with increasing *p*_*g*_. The RMSE was not affected by varying individual detection probability at *p*_*g*_ = 0.7 (Fig 3).

As an example of compared model performance, when *p*_*g*_ = *p*_*i*_ = 0.7, which are values comparable to those associated with the Roy *et al*. (26) real field dataset, the Two-Step model estimated a slightly higher population abundance than did the One-Step model (average means 535.7 versus 525.6, respectively; Table 1). All parameters returned by the Two-Step model were largely unbiased (3% or less) and exhibited nominal or almost nominal BCI coverage (Table 1), whereas detection probability estimates from the One-Step model showed moderate bias of 6% and both detection and abundance estimates had BCI coverage of <50%.

**Table 1:** Over 1000 population simulations, the average posterior mean, RMSE, coefficient of variation, % bias of the mean, and coverage of the 95% Bayesian Credible Interval of the estimates for overall population abundance, group size, number of groups, detection probability of groups (*p*_*g*_), and detection probability of individuals (*p*_*i*_) in the Two-Step and conventional CMR models (for capture histories simulated with *p*_*g*_ = *p*_*i*_ = 0.7).

	Average Mean	RMSE	Average CV	Average % Bias	Average BCI Coverage
**Two-Step CMR model**					
Total $\hat{N}$	535.68	44.37	0.08	3	0.942
Group size	13.05	0.62	0.046	0.3	0.954
Number of groups	41.15	3.35	0.078	3	0.966
*p*_g_*	0.68	0.075	0.103	-3	0.942
*p*_i_*	0.700	0.010	0.015	0	0.941
**Conventional CMR model**					
Total *N*	525.61	42.81	0.022	1	0.408
p*	0.677	0.087	0.030	6	0.332

*p_*g*_: group detection probability of gorilla groups with >1 members; *p*_*i*_: individual detection probability; *p*: individual detection probability not accounting for group structure of population

### Comparison of estimators with real data

The Two-Step model estimated a total of $\hat{N}$ = 285.4 (SD 35.74) gorillas, composed of 52.3 (SD 25.2) solitary individuals and 29.5 groups (SD 2.94) with, on average, 7.9 individuals (SD 0.61). Estimates of detection probability for groups, and individuals within groups, were similar (0.68 with SD 0.08, and 0.69 with SD 0.03, respectively), but detection probability of solitary individuals was much lower at 0.22 (SD 0.11). The conventional CMR model estimated a similar total $\hat{N}$ (279.4) but with erroneously higher precision (SD 20.58). Credible intervals for total $\hat{N}$ of both models widely overlapped. Detailed model output is summarized in Table 2.

**Table 2:** Posterior summaries (mean, standard deviation and 2.5^th^ and 97.5^th^ percentiles) for parameters from a Two-Step CMR model accounting for group structure (# groups, group size) and allowing single individuals never associated with a group (# solitaries) to have a separate detection probability from group-living individuals while estimating abundance (total $\hat{N}$), and from a regular CMR model that does not account for group structure in the population.

	Mean	SD	2.50%	97.50%
**Two-Step CMR model**				
Total $\hat{N}$	285.4	35.74	232	366
Group size	7.90	0.61	6.75	9.16
Number of groups	29.52	2.94	26	37
Number of solitaries	52.27	25.22	21	114
*p*_g_*	0.68	0.08	0.50	0.82
*p*_i_*	0.69	0.03	0.64	0.74
*p*_s_*	0.22	0.11	0.07	0.47
**Conventional CMR model**				
Total $\hat{N}$	279.43	20.58	245	325
p*	0.46	0.04	0.38	0.53

*p_*g*_: group detection probability of gorilla groups with >1 members; *p*_*i*_: individual detection probability conditional on group detection; *p*_*s*_: detection probability for solitary individuals; *p*: individual detection probability not accounting for group structure of population

## Discussion

Numerous examples exist where CMR approaches were applied to estimate the abundance of group-living species including: herding species such as elephants and pronghorn [4, 36, 37], pack species such as wolves and African wild dogs [13, 16], roosting species such as bats [38], aggregating species such as dolphins and whales [6, 39, 40], and grouping species such as chimpanzees and mountain gorillas [26, 41, 42]. Although some of these CMR studies described, to varying degrees, the potential dependence of individual-level detection on prior group-level detection, most CMR studies encountered by the authors ultimately assumed equal *p*_*i*_ of individuals [but see 41, 42] and none applied CMR approaches that explicitly addressed the dependent nature of detection probability in group-living species. A noteworthy exception was Marnewick *et al*. [16] who acknowledged that African wild dogs live in packs and therefore used the capture histories of the packs (not the individuals) to estimate the total number of packs in the study area. They then estimated mean pack size via count data from a tourist photographic survey to calculate the overall population estimate [16]. Analytical frameworks other than CMR have developed approaches to estimate abundance in group-living species. For example, distance sampling has been used to estimate the number of groups, then multiply group abundance with average observed group size to arrive at total abundance (e.g., [43]). This assumes, however, that group size is observed perfectly, and Clement et al. [32] expanded this approach to account for imperfect group size observations, using double-observer type group size data combined with N-mixture models. The general approach of combining models describing group and individual detection is thus a flexible one that can be adapted to different observation protocol.

Williams [44] explained the importance of accounting for uncertainty in wildlife conservation and management and provided a framework for coping with four basic types of uncertainty. CMR approaches attempt to reduce the uncertainty reflected in sampling variation in wildlife monitoring, often termed ‘partial observability’, by quantifying detection probability. Such variation can lead to uncertainty in population abundance or the status of the population. Here, we introduced a Bayesian CMR approach that incorporates group detection explicitly into the abundance-estimation procedure and resolves the issue of individual detection being dependent on group detection. Our results demonstrate that the simpler One-Step model falsely suggested high certainty in abundance estimates by ignoring the true hierarchical structure of the detection process. Even though this conventional CMR model produced, on average, unbiased estimates of abundance, BCI coverage was poor, especially when individual-detection probability was high. Moreover, maximum bias in any given scenario was higher for the One-Step model, in particular for low group-detection probability, suggesting that for a given data set the conventional model incurs a higher risk of stronger bias. The One-Step model also had considerably higher RMSE in scenarios with low group-detection probability. Misleadingly accurate abundance estimates could erroneously suggest significant differences between populations or, if replicated over time (as is done for the mountain gorilla population), could falsely suggest population trends when a population is, in fact stable.

Alternatively, the Two-Step model had high BCI coverage under all scenarios. The Two-Step model is more complex, therefore, its coefficient of variation (CV) tends to be higher than the simpler One-Step model, which may at first seem undesirable. However, our results suggest that the amount of uncertainty around $\hat{N}$ under the Two-Step model is a more accurate portrayal of the true uncertainty given the data and the detection process. Moreover, the Two-Step model provides additional information on population structure, by returning estimates of the number of groups and average group size. These quantities in themselves may be of ecological or conservation interest. Further, by separating out the two detection components, the Two-Step model opens up avenues for more specific modeling of variation in detection. For example, it is conceivable that variation in group-detection probability is more pronounced (due to group size or overlap of group territories with survey effort) than variation in detectability of individuals within that group, once a nest site is found. Finally, the Two-Step model also makes more efficient use of the available data, as it does not condense individual detections across multiple nest sites into a single binary variable per sweep. Our results suggested that data from three nest sites (sub-trials) were sufficient to produce highly accurate (bias <1%) estimates of group size, even when individual detection probability *p*_*i*_ was as low as 0.3 (much lower than *p*_*i*_ estimated at 0.69 from real data). Conversely, estimates of the number of groups showed some bias at low group detection probability *p*_*g*_. Again, real data analysis suggested that *p*_*g*_ for mountain gorillas was much higher, at 0.68. When surveying other group-living species with lower *p*_*g*_, however, it may be beneficial to invest more effort into detecting groups rather than individuals within groups.

Based on results here, we suggest that the Two-Step CMR approach be adopted for abundance estimation of mountain gorillas. This species is of extreme conservation concern and most studies in the last 20 years have concluded that mountain gorilla abundance has been increasing, without quantifying the simultaneous increases in survey effort over the same period. To draw conclusions about population status requires that we better estimate the uncertainty around any given population estimate such that we do not erroneously claim a trend, when in fact the population is simply stable. A robust estimate of the direction and magnitude of a trend, if any, will help managers assess the effectiveness of their conservation measures. Importantly, should a truly increasing population trend be detected for mountain gorillas, a re-assessment of carrying capacity will be critical, as quantity of habitat has remained static, at best, over the last several decades.

### Future research

The Two-Step CMR model developed in this study represents the simplest case scenario, where group sizes within the population show relatively low variability, group membership and size is constant within the study period, and detection probability is constant across groups and–within groups–is constant across individuals. Analogous to conventional CMR models, this model could easily be extended to incorporate variation in detection probability across time, captures and recaptures, or among groups/individuals (conventional models M_t_, M_b_ and M_h_, respectively; 2); or to include covariates on either group- or individual-detection probability. Particularly, group size may be an important predictor of group detectability [e.g., 45], as0 suggested by the much lower detection probability of solitaries in the present gorilla case study. In a Bayesian framework, estimated group size could readily be used as a covariate on group-detection probability (using an appropriate link function such as logit). When group size is an important determinant of group detectability, failure to account for this detection bias will likely lead to overestimated group size [45] and can lead to negatively biased estimates of the number of groups [23]. More variable group size could be modeled using more dispersed distributions; or, the model could reflect more complex social structuring of the population (for example, including the presence of solitary individuals in otherwise group-living species, as shown in the gorilla example). Alternatively, if individual-detection probability is sufficiently high once a group is detected, group size is observed perfectly and the model simplifies to one similar to Marnewick *et al*. [16], where the number of groups becomes the main focus of estimation. Finally, the hierarchical detection structure could readily be translated into a spatial capture-recapture framework, which accounts for heterogeneity in detection of groups due to differential exposure to sampling effort [22, 46]. It is worth emphasizing that the present model and all suggested extensions consider stable group memberships over the course of the study. Approaches that allow for a fission-fusion type population will likely be more challenging to develop. In such cases, it is conceivable that the added complexity necessary to describe group fluidity might outweigh the benefits of accounting for group structure.

## Conclusion

The current study represents an initial investigation into incorporating both group detection and individual detection into a single closed CMR approach to estimate the abundance and population structure of a group-living species. The main consequence of ignoring the dependence of individual detection on first detecting the group was severely overestimated precision, which in practice can translate into a misunderstanding of population abundance, trends, and status which can then lead to misguided conservation actions; this was combined with a higher risk for bias, especially at low group detection probability. We suggest that future population studies of species living in stable (i.e., negligible fission-fusion for the duration of a given study) groups consider the Two-Step approach when estimating total population abundance. Applications of this approach to field data, however, should incorporate important sources of variation in group- and/or individual-detection probability to obtain unbiased population estimates. Investigating the performance of the Two-Step model when detection is variable would be an important next step in further validating this novel approach.

## Supporting information

S1 Script R code for simulating data, fitting two-step and one-step models, and summarizing results. (R)

S2 Script JAGS code to fit conventional (One-Step) closed capture-recapture model. (TXT)

S3 Script JAGS code to fit Two-Step closed capture-recapture model. (TXT)

S1 Data All Individual Capture Histories for Gorillas Detected in Groups during Bwindi 2011 Surveys Summed by Sweep. (CSV)

S2 Data All Individual Capture Histories for Gorillas Detected as Solitary during Bwindi 2011 Surveys Summed by Sweep. (CSV)
